# Heuristic Pairwise Alignment in Database Environments

**DOI:** 10.3390/genes13112005

**Published:** 2022-11-02

**Authors:** Panna Lipták, Attila Kiss, János Márk Szalai-Gindl

**Affiliations:** 1 Department of Information Systems, ELTE Eötvös Loránd University, 1117 Budapest, Hungary; 2 Department of Informatics, J. Selye University, 945 01 Komárno, Slovakia

**Keywords:** pairwise alignment, bioinformatics, database systems

## Abstract

Biological data have gained wider recognition during the last few years, although managing and processing these data in an efficient way remains a challenge in many areas. Increasingly, more DNA sequence databases can be accessed; however, most algorithms on these sequences are performed outside of the database with different bioinformatics software. In this article, we propose a novel approach for the comparative analysis of sequences, thereby defining heuristic pairwise alignment inside the database environment. This method takes advantage of the benefits provided by the database management system and presents a way to exploit similarities in data sets to quicken the alignment algorithm. We work with the column-oriented MonetDB, and we further discuss the key benefits of this database system in relation to our proposed heuristic approach.

## 1. Introduction

Bioinformatics is an interdisciplinary field of molecular biology, genetics, computer science, mathematics and statistics [1]. As biology has evolved, ever larger data sets have become available to scientists, and computer science approaches have been used to process these data. The Human Genome Project, for example, discovered that human DNA is made up of 3 billion base pairs [2], but the project itself was made possible by modern bioinformatics tools. With the development of technology, larger data sets have become available during the last years. The new challenge is in processing them efficiently. This task requires the understanding of biology-related data, algorithmic aspects and, furthermore, knowledge of the advantages of different database management systems and architectures.

One important area in bioinformatics is the analysis of DNA sequences. From an algorithmic point of view, these bases are of paramount importance, and therefore, DNA sequences are defined as a sequence of bases in bioinformatics.

(1) $$DNA\;sequence\;=\;s_{1}s_{2}\ldots s_{n},\;n\geq 1,\;\forall s_{i}\;\in \;\left\{A,\;G,\;C,\;T\right\}$$

where *A* is adenine, *G* is guanine, *C* is cytosine and *T* is the equivalent of thymine. Sometimes other characters can occur in a sequence; for example, *N* indicates when sequencing has failed to determine which base is in a given position. Next-generation sequencing was established in the early 2000s. This technique does not read the whole genome at once, but only specific shorter regions, allowing for parallelization. The short DNA segments thus defined are called reads; the length of reads varies from technology to technology and is usually between 20 and 400 bases but can be up to 1000 bases long. The whole genome can be reconstructed from the reads. This is a splicing algorithm, where a reference genome is taken and the position of each read is searched, taking into account that the sequenced genome may contain mutations [3].

The comparison of two sequences is called pairwise sequence alignment and can be local or global. The latter means that whole sequences need to be aligned, which helps identify the evolutional relationship between them. Based on the alignment scoring system, an optimal alignment exist: the one with the highest score, in our case. The Needleman–Wunsch dynamic programming algorithm [4] finds the optimal alignment for any two sequences. In this version of the algorithm, each gap is scored with a fixed gap penalty, but if we take into account some biological characteristics, different gap penalties can be defined. For example, one longer mutational event is more likely than multiple shorter ones; therefore, in this study, we also examine affine gap penalty, which consists of a gap opening and a gap extending score. In the case of affine gap penalties, the optimal alignment can be found by Gotoh’s algorithm [5,6].

Advances in technology have created data sets of such size that storing and processing them has created new challenges for professionals. This is no different in biology, where a number of data warehouses have been created over the years (e.g., the National Center for Biotechnology Information [7]). Often, these types of databases are not only for local interests, but complex systems are created to make the data available in many parts of the world. An example of this is the emergence of specialised sequencing databases (e.g., GISAID [8]) in the context of the 2019 coronavirus outbreak because it was in the global interest to publish and share the viruses sequenced in different countries.

There are many advantages to storing biological data in databases. For example, these systems usually have built-in features for managing different access rights, concurrent access or even data encryption. Fast, complex searches can be implemented by indexing up-to-date and expanding data sets. The question that we have been exploring is whether it is possible to efficiently perform pairwise alignment in a database environment where the sequences are already stored. Within the database, there are optimised representations of the data as well as optimised operators and functions for the different structures. With data-intensive problems, a common approach is to move the algorithm to the data, not vice versa, to reduce I/O operations.

Given a database system as the environment, subresults can be stored during algorithm execution. Our idea was to store relatively good subalignments from previous alignments to build a knowledge base and to use them for upcoming alignments. For the subalignments, we take the optimal alignments of each pair from a randomly chosen sample set and split the representative strings into substrings. These are stored in a table and queried for suitable candidates if a new alignment needs to be performed. If the best candidate is found, it can be inserted into the alignment, which reduces the size of the problem significantly. After inserting subalignments, the remaining unaligned parts are aligned with a dynamic programming algorithm. Our measurements showed that with choosing the appropriate parameters, the algorithm outperforms the original solutions in time and gives a close-to-optimal result.

## 2. Materials

Pairwise alignment may be the foundation of other important algorithms such as multiple sequence alignment. The development of methods for multiple alignment is an area of active research. For example, [9] presents automatable methods to correct some common alignment errors. Further, Korotkov et al. [10,11] present new mathematical methods that can cope with cases where the average number of substitutions per position in the sequences is above a certain threshold, in contrast to previous work.

Over the years, a number of different approaches have been published to improve pairwise alignment. First of all, we introduce the original solution: the Needleman–Wunsch dynamic programming algorithm [4]. Given two sequences *A* and *B* of length *n* and *m*, respectively, this method creates a matrix of the size (n+1)×(m+1). The initialization of this matrix can be seen in Equation (2), and evaluation of the cells is seen in Equation (3), where i=1…n, j=1…m and *c* is the scoring function for any two characters of the sequences or gap symbols (–). In this paper, we used score instead of distance; so in this case, matching characters have a positive value, while mismatches or gaps have a negative value.

(2) $$F\left[0,0\right]\;=\;0,\;F\left[i,0\right]\;=\;i\cdot c\left(a_{i},\;‘-’\right),F\left[0,j\right]\;=\;j\cdot c\left(‘-’,b_{j}\right)$$

(3) $$F\left[i,j\right]\;=\;\max\left\{\begin{array}{c} F\left[i\;-\;1,j\;-\;1\right]\;+\;c\left(a_{i},b_{j}\right)\\ F\left[i\;-\;1,j\right]\;+\;c\left(a_{i},‘-’\right)\\ F\left[i,j\;-\;1\right]\;+\;c\left(‘-’,b_{j}\right) \end{array}\right.$$

To define the optimal alignment, a backtrack phase should be performed. When a cell gets a value, the case that gave the maximum needs to be stored, and this would be marked as the previous cell. From the F[n,m] cell, optimal alignment can be defined from behind by following this backtrack route. The complexity of the algorithm is O(nm). For an affine gap penalty, the gap scoring function is shown in Equation (4), where $k\in N^{+},\;gap_{opening},\;gap_{extending}\;<\;0$.

(4) $$g\left(k\right)=gap_{opening}+k\cdot gap_{extending}$$

Gotoh’s algorithm [5,6] provides optimal alignment with affine gap penalties. The solution requires multiple matrices for evaluation (*F*, *P*, *Q*) and backtracking. The initialization of the matrices is shown in Equation (5), and the filling of the matrices is in Equations (6)–(8). In the backtracking phase, a step may not only change the current cell but also the current matrix. The complexity of the algorithm remains O(nm).

(5) ∀i=0…n,∀j=0…m:F[i,0]=g(i),F[0,j]=g(j),P[i,0]=Q[0,j]=−∞

(6) $$F\left[i,j\right]=\max\left\{\begin{array}{c} F\left[i-1,j-1\right]+c\left(a_{i},b_{j}\right)\\ P[i,j]\\ Q[i,j] \end{array}\right.$$

(7) $$P\left[i,j\right]=\max\left\{\begin{array}{c} F\left[i-1,j\right]+gap_{opening}+gap_{extending}\\ P\left[i-1,j\right]+gap_{extending} \end{array}\right.$$

(8) $$Q\left[i,j\right]=\max\left\{\begin{array}{c} F\left[i,j-1\right]+gap_{opening}+gap_{extending}\\ Q\left[i,j-1\right]+gap_{extending} \end{array}\right.$$

In the following, some heuristic algorithms are presented that solve pairwise sequence alignment with less complexity but are not guaranteed to give the optimal solution, only to approximate it sufficiently. Bounded dynamic programming [12] is a possible method to solve the problem of the large storage and time requirements of the original algorithm. In pairwise alignment of similar sequences, we assume that the optimal result does not contain many gaps. Therefore, the backtracking remains close to the diagonal of the matrix. The algorithm based on this observation is called k-band alignment, where values are computed only *k* distance in each direction from the diagonal of the matrix, i.e., the cell F[i,j] is assigned a value if −k≤i−j≤k. If *k* is larger, the computational demand increases, but the probability of finding the optimal solution also increases. For smaller *k* values, the number of operations performed can be significantly reduced. The time requirement for k-band alignment is O(kn), where *n* is the number of bases in the longer sequence.

**Figure 1:**
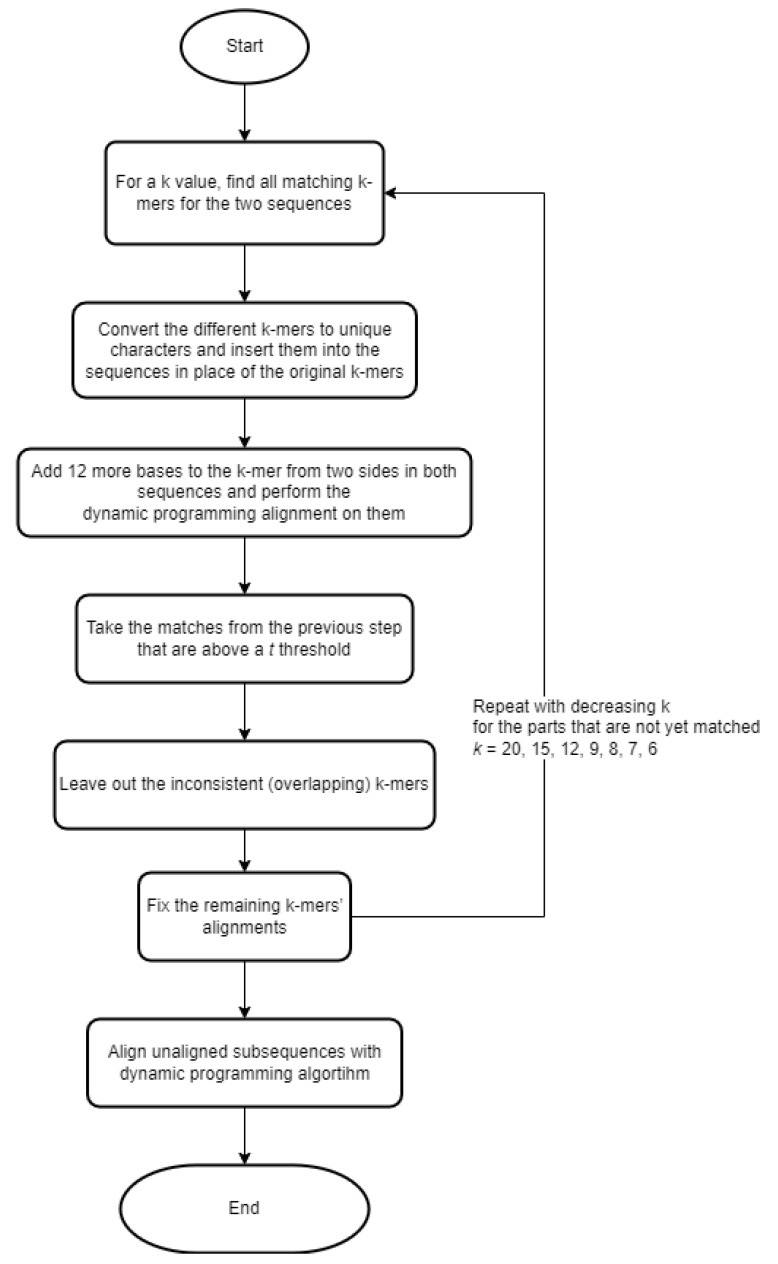
Flowchart of the GLASS steps.

The MUMmer [13] algorithm tries to identify similar regions for large sequence sets. MUM stands for maximal unique match, i.e., the maximum (non-extensible) matching subsequence that occurs only once between two sequences. The concept is that a MUM is almost certainly part of the global alignment, and therefore, the entire match can be built around it. To find MUMs, the algorithm generates suffix trees, a data structure often used in solving pattern-matching problems, as they can be generated and searched in linear time. When the algorithm has found the MUMs between two sequences, it sorts the MUMs according to their starting positions in one of the sequences. Since there may be cases where the starting positions of the MUMs ordered in this way are not in (completely) ascending order in the other sequence (e.g., two consecutive MUMs in one sequence appear in reverse order in the other sequence), and global alignment is the goal, we want to take a subsequence of the starting positions taken from the other sequence that is as long as possible. For this task, MUMmer uses a variant of the longest increasing subsequence algorithm (LIS) [14]. GLASS [15] (GLobal Alignment SyStem) implements an iterative algorithm. This method is based on the use of k-mers. The steps are briefly represented in a flowchart (Figure 1).

In recent years, the use of artificial intelligence methods to solve complex problems has become increasingly common. In the case of pairwise alignment, an example is DQNalign [16]. This approach is based on deep reinforcement learning, whereby an agent must learn to make appropriate decisions in a given environment to achieve the desired outcome. Due to the complexity of the problem space, traditional reinforcement learning has proven to be insufficient. The essence of the method is to examine pairs of subsequences within a certain window instead of the whole sequence. The agent can perform three possible actions: go forward, insert, and delete. To score the actions, it uses the scoring of the alignment. During preprocessing, it searches for the longest common subsequence, or MUM, to determine the algorithm’s starting point as favourably as possible. DQNalign results in high accuracy and low complexity with an appropriate adaptive window size, but there are some datasets on which MUMmer performs better. The combination of AI algorithms with heuristic methods may offer further potential for improvement.

## 3. Method

In this section, the proposed heuristic alignment and the chosen database environment are presented. The method is applicable for cases where a large set of similar sequences needs to be aligned. We exploit this similarity by storing high-scoring subalignments in the database and using them for the upcoming alignments, thereby decreasing the size of the original problem.

For the database environment, we have chosen MonetDB [17]. This relational database management system has been developed by CWI since 1993. Its various characteristics make it a good candidate for handling and processing large data sets. Some of these features are:

- Column-oriented: records are not stored by row; they are split vertically and each column is stored in a separate table. This is beneficial because a column contains one data type; therefore, data compression can be applied. On the other hand, in databases, we think in entities, where one row represents one entity and its attributes are stored together, while in a column-oriented database, for example, on insertion, each attribute is written in a different table and physically in a different space on the disk. Additionally, reconstructing a row means joining the columns together every time. A row-oriented database reads all of the attributes and discards the ones we do not need, while the column-oriented version only reads the necessary attributes [18]. In a paper in 2015, MonetDB and PostgreSQL were compared for storing and processing variant data, and MonetDB not only used less memory but also ran the proposed queries faster on average [19]. In our case, a column-oriented database is an appropriate choice because it performs well in read-intensive usages. Write and update operations make up smaller proportions or are performed periodically in our proposed approach.
- Main-memory/in-memory: In contrast to disk-resident databases, main-memory databases load the data into the RAM and access it from there. Reading from the disk is a much slower process, and nowadays, having more RAM is an affordable option. If the data do not fit into the main memory, then the database utilizes virtual memory and swapping [20].
- Adaptive indexing: Occasionally, when handling large data sets, there is not enough a priori knowledge to define optimal indexes. This can be solved by adaptive indexing. Without human intervention, it creates indexes based on the previously run queries. It is possible to define indexes, but the optimizer disregards them if a more efficient solution is available. MonetDB uses a secondary-cache-based index [21].
- Open source: MonetDB’s entire source code is available on GitHub [22]. It is under continuous development, and the development team is quick to handle bugs and answer questions.
- Embedded Python: When defining a function in MonetDB, it is possible to set the language to Python. This means that the input parameters are converted to NumPy types and the body of the function is Python code. This eliminates the need for an external interface to perform operations on the data.

MonetDB has been used several times in the literature to store and process biological data [23,24]. Based on the aforementioned features of MonetDB, it is a proper candidate to solve pairwise alignment in a database environment. For example, as the sequence data are not updated, this satisfies the read-intensive use case.

**Figure 2:**
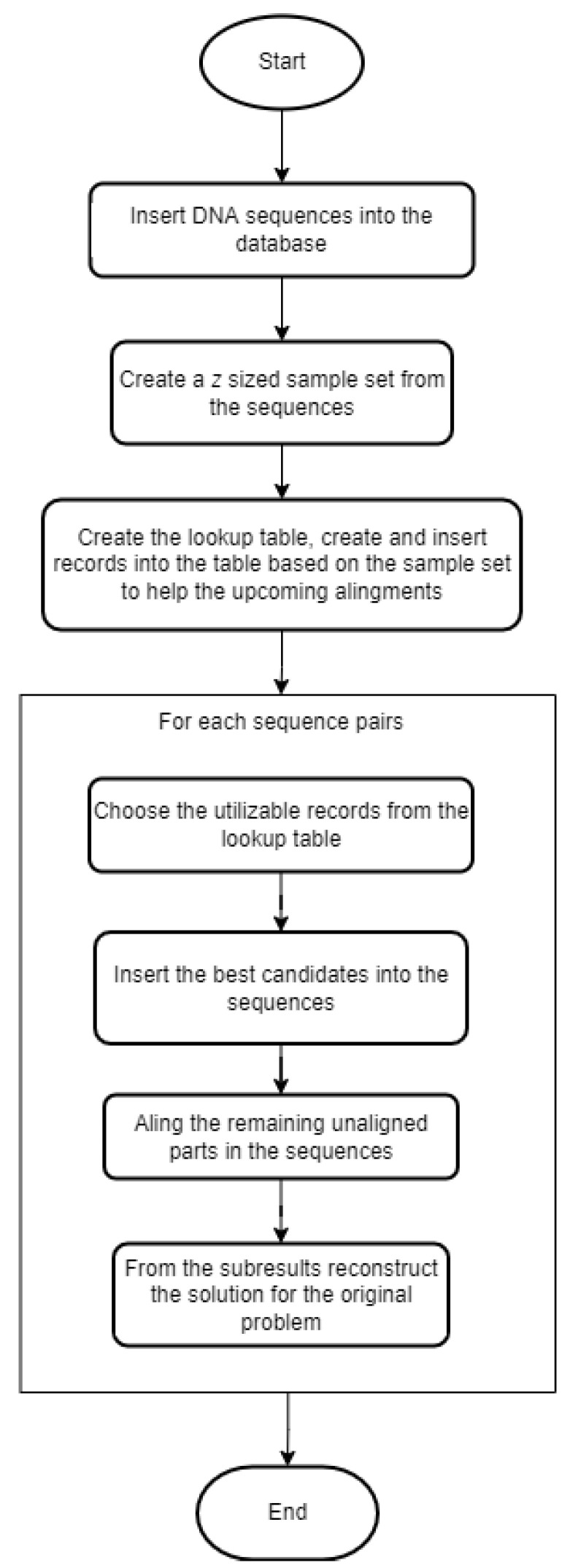
Flowchart of our heuristic algorithm steps.

Before presenting the proposed method in detail, we need to define the problem that we want to solve: a large set of similar sequences are given, and we want to run pairwise alignment on all possible pairs to determine the relationship between them. This could be the foundation for creating phylogenetic trees or multiple sequence alignments. The steps of our heuristic algorithm are represented in a flowchart (Figure 2).

These are the schematic steps of the algorithm. In the next couple of paragraphs, we present them in greater detail.

Step 1 is inserting DNA sequences into the database. For this, a table needs to be defined with at least two attributes: id as a primary key, and seq is a text field that contains the actual string of the bases. Those are the necessary columns, but naturally, the table can be extended with further attributes if needed. We refer to this table as seq_data.

Step 2 is generating a *z*-sized sample from the previously inserted sequences. With MonetDB, this can be easily achieved because it provides a keyword at the end of a query called ‘SAMPLE’. In an expression after the keyword, the size or the proportion of the sample can be defined. This keyword cannot be parameterized; therefore, a small workaround is needed: we define a function with Python to construct and run the sampling query with any given numeric parameter. We store the sequences that are chosen to be in the sample set in a table called sample_seq.

Step 3 is creating and inserting records into the lookup table. First, we take the sequences from the sample set and generate all possible pairs. For each pair, we run the Needleman–Wunsch algorithm, which results in the optimal pairwise alignment. This alignment is represented in two strings, and from these strings, all pairs of overlapping k-mers are generated. From these k-mers, we only keep the ones that are above a given threshold score, *k*. The threshold and the scoring system are the input parameters for this step. The remaining k-mers are the subalignments we want to reuse in the upcoming alignments.

The subalignment strings stored in the lookup table may contain gap symbols. However, when we want to use these strings for pairwise alignment, the sequences that make up the pair will obviously not contain such characters. Therefore, the search should somehow remove them. Suppose, for example, that we have a stored subalignment: (‘AC–A’, ‘ACCA’). If one of the sequences contains the subsequence ‘ACA’ and the other contains ‘ACCA’, then the stored subalignment can be utilized. To avoid the need to always remove the gap symbols, we store the base strings to be searched for in a separate table called aligned_subseq. This also allows us to decrease redundancy: ‘–ACA’, ‘A–CA’, ‘AC–A’, ‘ACA–’; ‘ACA’ subalignment strings can be all bind to ‘ACA’ in the aligned_subseq table. In this table, each subsequence is given an id, and in the lookup table, the records refer to those ids which help in the searching phase.

Although there are sequence analysis programs that handle degenerate bases, namely the unspecified or unknown base *N*, in the same way as for the *A*, *C*, *G* or *T* bases (with the same match and mismatch scores), most of them do it differently, e.g., BWA-MEM [25]. Kim and Park also argue for special treatment of *N* in their work [26] and introduce an ‘unknown’ score (e.g., with a value of zero, but not necessarily), which is given if at least one of the two bases to be compared is *N*. If this approach is followed, the question arises as to how to handle subalignment strings where there are *N*s. There can be a number of ways to do this, which are beyond the scope of this paper, but one possible approach is outlined. For example, if one string contains *N* base(s) (e.g., ‘ANN’), then in addition to the string containing *N*, we can enter the bases *A*, *C*, *G* or *T* in all possible ways in the positions containing *N* and store the resulting strings in the aligned_subseq table (i.e., in addition to ’ANN’, the strings ’AAA’, ’AAC’, …, ’AAN’, ’ACA’, …, ’ANT’ are also stored) so that they can be used for further pairwise alignments.

After filling the lookup and the aligned_subseq tables with records, the remaining pairwise alignments can be performed, which is Step 4. For each pair, the subalignment candidates must first be selected, i.e., for both sequences it must be determined which of the subsequences in aligned_subseq table they contain. The result of this query is the ids of the records and the indices where the subsequences begin in the original sequence, and for the next product, the result is sorted by the ids of the records in ascending order. Once we have this information for both sequences, we take the product of the results of the two queries and select the pairs of ids present from the lookup table. Here it should also be indicated which id belongs to which sequence, and this can be achieved, for example, by specifying a constant expression as an element of the select clause that makes up the query. The results are sorted by score because we want to start with the candidates with the highest scores.

By completing the previous steps, we get the subalignment candidates for the given two sequences, supplemented by the indices on which the subsequences begin and the order of the sequences. At this point, we apply a filter: if the two start indices are too far away, then we can assume that many multiple gap symbols need to be inserted before the subalignment, which could result in an overall unsatisfactory alignment; therefore, we discard these candidates. The threshold we used was the following in the case of a fixed gap penalty: $-|ind_{1}-ind_{2}|*score_{gap}<score$, and the following in the case of an affine gap penalty: −(|ind1−ind2|·score_gap_extend+score_gap_open)<score.

From the remaining subalignment candidates, we always choose one with the highest score and check if it can be inserted in the alignment. It can be inserted if it is not overlapping with any previous subalignment. For this, we store an array of the size of each sequence, and if a subalignment is inserted between positions *p* and *q*, then we change the array values in these positions. Note that r=q−p≤k because the affected positions are the bases, so *r* is the length of the subalignment string without gap symbols. We can check if an upcoming alignment is overlapping by checking the values of the arrays, and if it is overlapping, then we discard it.

**Figure 3:**
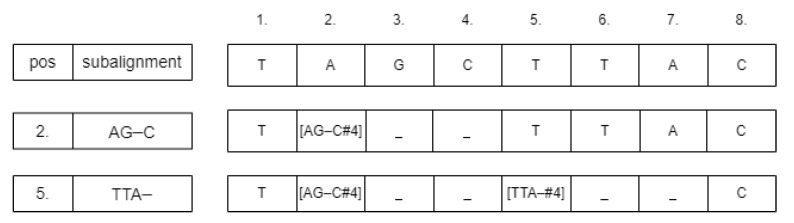
Example of inserting subalignments of length 4 into a sequence.

For inserting the subalignment, we convert the sequence strings into arrays. If the starting position is *p*, then we change the value of the array at *p* according to the following schema: [<subaligment>#<score>]. The values between p+1 and p+1−r need to be neutralized, for example, by changing them to the ‘_’ symbol. Figure 3 shows an example of this process. The neutralizing approach was chosen because deleting those cells could change the calculated starting positions of the subsequences. After inserting the subalignments, there could be some parts of the sequences that are unaligned. We detect those parts and run the Needleman–Wunsch alignment algorithm on them. From these subresults, the whole global pairwise alignment can be reconstructed.

**Figure 4:**
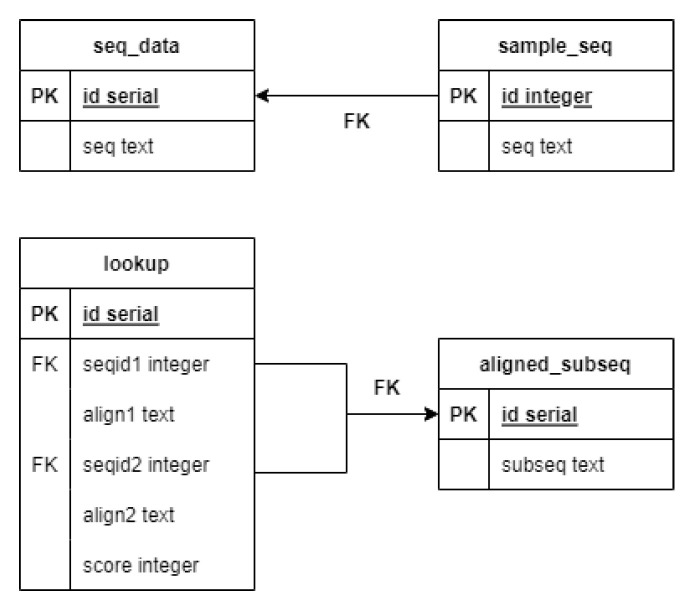
The tables used in the heuristic pairwise alignment.

By executing the steps described above, a heuristic pairwise alignment can be constructed. Figure 4 shows the description and relations of the tables used for the algorithm. Our analysis showed that this method can result in close-to-optimal alignment in less time than the original dynamic programming algorithm.

### Asymptotic Complexity

The proposed method is a heuristic approach: it is not guaranteed to find the optimal alignment in each case, but with the right configurations, the result is close to optimal. The process can be divided into two phases: pre-processing and alignment.

The pre-processing phase is when the lookup table is filled with records. This can be performed periodically. For example, if sequences are stored for a specific virus and the data set expands with new data every week, then performance of the heuristic method should be monitored, and if the quality decreases with the new data, then the lookup table needs to be updated accordingly. We handle the complexity of this phase separately because it can be done between alignment phases, and if we store the previously run alignment results, then that information can be used to speed up this operation. In the Results section, we present how much time this operation takes with different parameter settings.

Therefore, we only examine the time complexity of the alignment phase in more depth. Let $h_{avg}$ be the average length of the sequences in the data set, *k* the length of the k-mers and *z* the size of the sample set. If we take a sequence pair and perform the alignment, then it can be split into $O(h_{avg})$ k-mers. This is for one pair, but the sample set can produce $\frac{z\cdot (z-1)}{2}=O\left(z^{2}\right)$ pairs. Thus, the sizes of the lookup and aligned_subseq tables are $O(z^{2}\cdot h_{avg})$. Another approach to the size of the aligned_subseq table could be as follows: it stores the unique gapless versions of the k-mers, so the length of these are r≤k. If the characters can only be the 4 bases, then the size of this table can be estimated as $\sum_{r=1}^{k}4^{r}=4\cdot \frac{\left(4^{k}-1\right)}{3}=O\left(4^{k}\right)$. However, this can only be a narrower upper bound if *k* is small. (For example, for k=6, this value is 5460, but for k=7 it is 21,844, and the other estimate is around 15,000 for z=10, $h_{avg}=150$).

For every subsequence, a pattern-matching algorithm needs to be performed. The best case for single pattern matching is $O(h_{avg})$, and the worst case is $O(k\cdot h_{avg})$ based on differences in the implementation. Considering the number of subsequences, we get $O(z^{2}\cdot h_{avg}^{2})$ and $O(k\cdot z^{2}\cdot h_{avg}^{2})$ complexities for this step of the algorithm. Subsequences from the aligned_subseq table that occur in a sequence have their ids ordered. Since MonetDB uses TimSort (see https://github.com/MonetDB/MonetDB/blob/master/gdk/gdk_batop.c and https://github.com/MonetDB/MonetDB/blob/master/gdk/gdk_ssort_impl.h, accessed on 11 October 2022), its performance is $O(z^{2}\cdot h_{avg}\cdot \log\left(z^{2}\cdot h_{avg}\right))$ [27]. After we determine which subsequences are present in the sequences, we have to identify the records in the lookup table that can be used. MonetDB is described as self-optimizing and creates index structures based on the queries it runs. It is assumed that since this is a frequently used query, the lookup table has indexing, i.e., it is ordered. Thus, the cost of the JOIN operation is $O\left(z^{2}\cdot h_{avg}+z^{2}\cdot h_{avg}\right)=O\left(z^{2}\cdot h_{avg}\right)$ due to the cost of merging two ordered tables [28].

If there is no subalignment for a pair of sequences, then the complexity of the alignment step is $O(h_{avg}^{2})$. If there is one subalignment that can be inserted in position *p*, then the complexity changes as follows: the alignment of the subsequence before position *p* is $O(p^{2})$; for the remaining subsequence after *p*, it is $O({\left(h_{avg}-p-k\right)}^{2})$; and the insertion itself is O(k). In this case, every component is less complex than the original dynamic programming solution, so the complexity of the alignment step in the proposed algorithm is less than or equal to that of the Needleman–Wunsch algorithm. The complexity of the whole heuristic approach is $O(k\cdot z^{2}\cdot h_{avg}^{2})$. This contains the extreme cases, which are unlikely if the method has been given reasonable input parameters. In practice, this heuristic algorithm performed well on the examined data sets.

As mentioned above, if we want to prepare our system for the presence of *N* bases, it becomes more complicated to handle subalignments. If we follow the approach given earlier as an example, then the estimation requires that the proportion of *N* within a sequence is denoted by $N_{rate}$ ($0<N_{rate}<1$). The value of $N_{rate}$ may depend on several factors and may vary between use cases [26], but in any case, it is true that the same rate is valid for k-mers (on average). Thus, instead of one k-mer, on average, $5^{k\cdot N_{rate}}$ are produced, because the number of *N*s within a k-mer is, on average, $k\cdot N_{rate}$. For this reason, taking a sequence pair from the sample set and performing the alignment, we can conclude that it can be split into $O(h_{avg}\cdot 5^{k\cdot N_{rate}})$ k-mers. So in this case, the sizes of the lookup and aligned_subseq tables can be estimated by $O(z^{2}\cdot h_{avg}\cdot 5^{k\cdot N_{rate}})$. On the other hand, the aligned_subseq table can also be estimated with $O(5^{k})$, but then it will still be true that, given meaningful numbers, $O(z^{2}\cdot h_{avg}\cdot 5^{k\cdot N_{rate}})$ is the sharper estimate. So in total, the complexity of our heuristic approach would be $O(k\cdot z^{2}\cdot h_{avg}^{2}\cdot 5^{k\cdot N_{rate}})$ for the case with *N* bases.

## 4. Results

To measure the performance of the algorithm, two types of data sets were used: generated and real sequences. The generated sequences were created by the Rose [29] software using the Jukes–Cantor evolutional model [30]. Other important input parameters are presented in Table 1. We measured how the parameters scaled with the length of the sequences and how the diversity between sequences affects the results.

The real sequences were Porcine circoviruses [31] from the NCBI database [7]. Each data set contained 100 sequences (which means 4950 global pairwise alignments were performed), and for each parameter setting, the heuristic algorithm was run 5 times and the results were averaged because the method is not deterministic (it contains random sample-set generation).

The input parameters of the method are:

- *k*: length of the subalignments;
- *z*: size of the sample set;
- score_match,score_mismatch,score_gap (open/extend): scoring scheme of the alignments. We used 1,−1,−2 for the fixed gap penalty (which is a commonly used choice [32]) and −3,−1 for the gap opening and extending penalties;
- threshold: subalignments are stored if they are above this score. We determined it based on the *k* value: $threshold=\lceil \frac{k}{2}\rceil \cdot score\_match$.

We used MonetDB v11.44.0 installed from the source. The implementation contains SQL and Python codes and is available at [33]. For comparison, we also implemented the Needleman–Wunsch and Gotoh dynamic programming algorithms, which always give the optimal alignment; we refer to them as the original algorithms.

### 4.1. Standard Deviation

We measured the goodness of the heuristic algorithm with the standard deviation of the alignment scores: let $score_{H}$ be the heuristic score and $score_{O}$ the optimal alignment score for one pair of sequences; the standard deviation for *n* pairs is $\frac{\sum_{1}^{n}{\left(score_{H}-score_{O}\right)}^{2}}{n}$. It is important to note that we only included the pairs in this calculation where the heuristic alignment was utilized.

**Figure 5:**
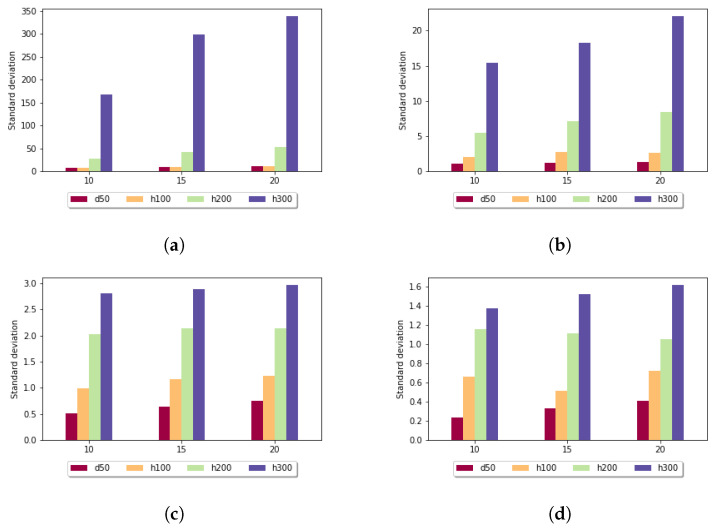
Standard deviation (y axis) depending on the size of the sample set (x axis) and length of the k-mers in the case of fixed gap penalty: (**a**) *k* = 5; (**b**) *k* = 7; (**c**) *k* = 10; and (**d**) *k* = 15.

Figure 5 shows the standard deviation for the fixed gap penalty case. The diagrams differ in the length of the k-mers. When *k* was set to 5, the standard deviation varied over a large scale: for sequences of length 100, it was around 9, but for longer sequences, it went up to 268. For larger *k* values, the imbalances were diminished; for example, in the case of k=10 in all data sets, the measured values were under 3. It can be stated that the size of the sample set did not have a significant impact on these results.

**Figure 6:**
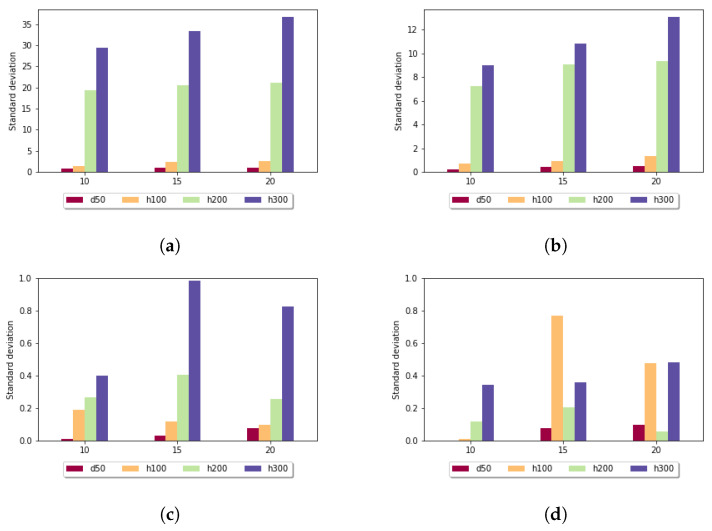
Standard deviation (y axis) depending on the size of the sample set (x axis) and length of the k-mers in the case of affine gap penalty: (**a**) *k* = 7; (**b**) *k* = 10; (**c**) *k* = 5; and (**d**) *k* = 15.

Figure 6 shows the standard deviation for the affine gap penalty. A similar tendency can be observed, because in this case also, longer subalignments resulted in smaller standard deviation values. For k=10,15 in all data sets, the measured values were under 1. It is also noticeable that for each parameter setting, the affine gap penalty had smaller deviation.

### 4.2. Utilization Rate

The heuristic approach can be used if there is at least one record in the lookup table that can be utilized for a given pair of sequences. Therefore, it was important to examine that how many times this occurred. We present these results as a percentage.

**Figure 7:**
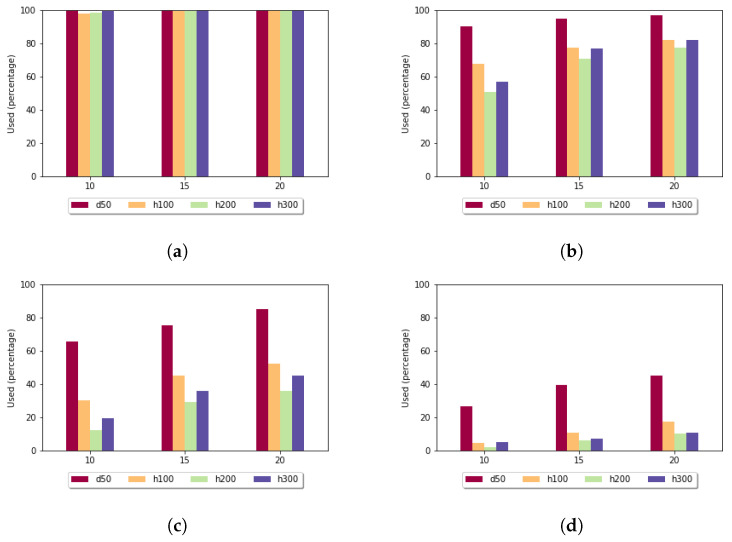
Utilization rate (y axis) depending on the size of the sample set (x axis) and length of the k-mers in the case of fixed gap penalty: (**a**) *k* = 5; (**b**) *k* = 7; (**c**) *k* = 10; and (**d**) *k* = 15.

Figure 7 shows the utilization rate in the case of the fixed gap penalty. For k=5, for almost each pair of sequences, the heuristic alignment could be used. By increasing the length of the subalignments, this rate decreased. For the more-diverse data sets (h100, h200, h300), in the case of k=15, it was less than 20%. As expected, the utilization rate was the highest for the least-diverse data set (d50).

**Figure 8:**
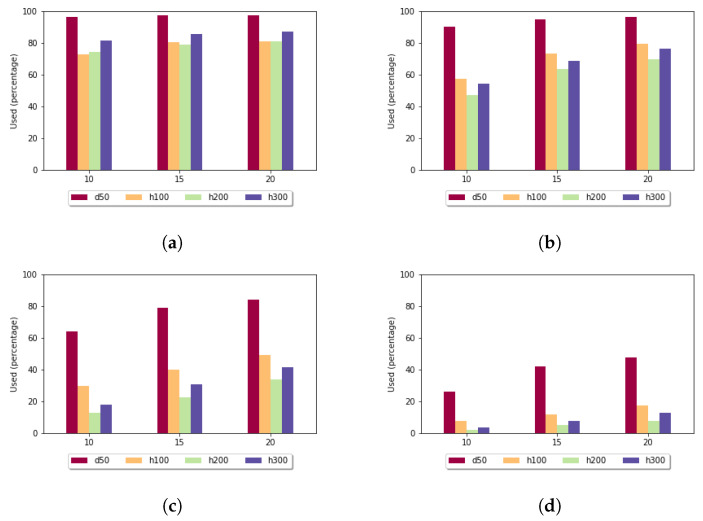
Utilization rate (y axis) depending on the size of the sample set (x axis) and length of the k-mers in the case of affine gap penalty: (**a**) *k* = 5; (**b**) *k* = 7; (**c**) *k* = 10; and (**d**) *k* = 15.

Figure 8 shows the utilization rate in the case of the affine gap penalty. Here it was even more significant that the least-diverse data set produced the highest utilization rate. For sequences of length 300 and if *k* was set to 15, the size of the sample set had an impact: for size 10, the utilization rate was 3.79%, while for size 20, it rose to 12.68%, which means that by doubling the number of sequences in the sample set, the examined value increased 3.35 times.

### 4.3. Exact Match Rate

We also examined how many times the heuristic approach found the optimal alignment. These percentages are presented in the following graphs.

**Figure 9:**
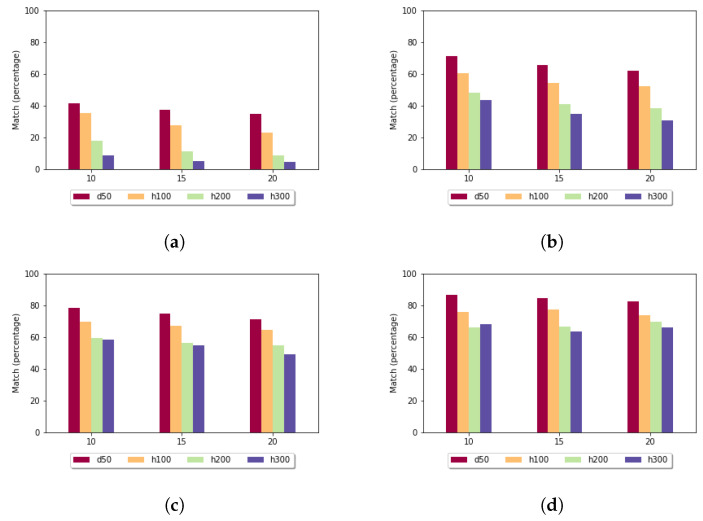
Exact match rate (y axis) depending on the size of the sample set (x axis) and length of the k-mers in the case of fixed gap penalty: (**a**) *k* = 5; (**b**) *k* = 7; (**c**) *k* = 10; and (**d**) *k* = 15.

In the case of the fixed gap penalty (Figure 9), if the *k* parameter increased, then the exact match rate increased. In the data sets with higher diversity and longer sequences, the optimal alignment was found at a lower rate. The presented results are connected to the standard deviation: when it was low, the exact match rate was high.

**Figure 10:**
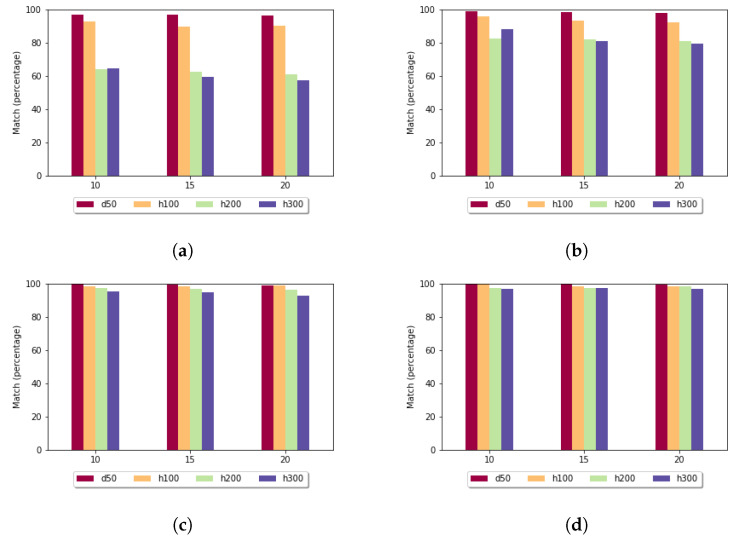
Exact match rate (y axis) depending on the size of the sample set (x axis) and length of the k-mers in the case of affine gap penalty: (**a**) *k* = 5; (**b**) *k* = 7; (**c**) *k* = 10; and (**d**) *k* = 15.

When the affine gap penalty was used (Figure 10), then, on average, the exact match rates were higher compared to the fixed gap penalty. If *k* was set to 10 or 15, then the heuristic method found the best alignment in almost every case when it was usable.

### 4.4. Run Time

A heuristic approach is adequate if it achieves a close-to-optimal result quickly. Therefore, the run times of the algorithms were measured for each data set with different *k* values. The size of the sample set had no influence on the run time, and we measured the pre-processing phase separately. In the following tables, *time*${}_{H}$ is the run time of the proposed heuristic approach, *time*${}_{O}$ is the run time of the original algorithm, *diff* is the difference between them, and *diff*${}_{\%}$ is the difference in percentage (*diff*${}_{\%}$$=(1-\frac{{\mathrm{time}}_{H}}{{\mathrm{time}}_{O}})*100$). For each case, the heuristic algorithm was faster than the original algorithms.

In the case of the fixed gap penalty (Table 2), for data sets d50 and h100, the run times were almost identical for each *k* parameter setting, and on average, the difference was around 45.44%. For longer sequences, the difference became more significant: for h300, it was 60.01% on average.

For the affine gap penalty, even the original algorithm took more time (it uses multiple matrices). The results shown in Table 3 have an average difference of 50–60%. If we examine data sets h100 and h300, we observe that the two algorithm scaled similarly: both of the run times increased eightfold for the longer sequences.

Despite the complexity estimation presented in the previous section, the proposed heuristic approach was faster for each data set.

### 4.5. Pre-Processing Time

Inserting subalignment records into the lookup table is considered pre-processing, which is not strictly part of the run time of the heuristic algorithm. However, it is important to note how much time this phase takes, since it needs to be performed periodically. Table 4 and Table 5 show the measured results for the two gap penalty cases. The run time was not significantly influenced by the size of the subalignments (*k*); therefore, they are presented by sample size. Increasing the sample set size significantly affected the time taken to populate the table: for 10 sequences, 45 pairwise alignments need to be performed, while for 20 sequences, this number is 190.

If we add the pre-processing time to the run time of the heuristic algorithm, we can calculate how many alignments the method needs for it to be paid off completely. For example, in the case of data set h100 and using 10 sample sequences, if the method can be used for at least 37 pairs of sequences, then the 1.6355 s pre-processing time is paid off, and the rest is pure gain in run time.

In the case of the affine gap penalty, the pre-processing took more time since the original alignment algorithm is more complex. Another example: for data set h300 and 20 sample sequences to gain time, the heuristic approach needs to be utilized 340 times, and our measurements showed that for k=15, it took 627 times on average.

### 4.6. Comparison with a Classical Algorithm

Using the algorithm inside the database environment has many advantages; however, the question arises of how efficient it is compared to a classical global pairwise algorithm. For the comparison, we chose the Bioconductor [34] R package; more precisely, we chose the *pairwiseAlignment* function from BioStrings [35]. This function implements the Needleman–Wunsch algorithm. Hereafter, we refer to it as Bioconductor for simplicity. We performed two experiments with Bioconductor: in the first one, the sequences were read from a local file; in the other one, they were queried from the database. Table 6 shows the time and peak memory usage of the classical algorithm and the proposed heuristic approach in the case of the fixed gap penalty averaged over 5 runs and using the same scoring system. The heuristic method was used with 10 samples and k-mers with a length of 10. The memory usage entails acquiring the sequences by reading them either from the database or from a file.

The classical algorithm with file reading can construct the alignments in less time by approximately one and a half orders of magnitude, although its memory usage scaled with the length of the sequences more significantly, while the heuristic approach scaled less than linearly. Reading the sequences from the database added a significant overhead in time compared to reading from a local file. It is important to note that peak memory monitoring also contained the creation of the lookup table, but even so, it used less RAM than the other two methods.

### 4.7. Real Data Set

Creating data sets allowed us to observe how the characteristics of the input affects the performance of the algorithm, and it has achieved positive results. However, the important question is whether this improvement can be reproduced in a data set that contains real sequences.

In the following, we present the measured results for a real data set containing Porcine circovirus sequences. It contained a hundred sequences, and the average length was 1642 bases. The length of the subalignments was set to 300, and the sample set contained 5 sequences. This parameter setting was chosen based on the observation of the previously shown results. The results are the average of 5 runs.

Results for fixed gap penalty:

- Standard deviation: 0.1073.
- Average utilization rate: 31%.
- Average exact match rate: 97.16%.
- Average run time for one alignment with the heuristic approach: 1.708 s.
- Average run time for one alignment with the original algorithm: 7.476 s.
- Average pre-processing time: 154.476 s.

For comparison, the highest alignment score was 1937. The heuristic method took 77.15% less time and it proved to be sufficiently accurate since the standard deviation is relatively low while the exact match rate is high. Although the heuristic approach was utilized in fewer than half of the pairs, the run time difference of the two algorithm was 2.5 h.

Results for affine gap penalty:

- Standard deviation: 0.
- Average utilization rate: 26.19%.
- Average exact match rate: 100%.
- Average run time for one alignment with the heuristic approach: 6.33 s.
- Average run time for one alignment with the original algorithm: 25.72 s.
- Average pre-processing time: 319 s.

In the affine case, if the algorithm found at least one subalignment that it could use, it found the optimal alignment. However, only in about a quarter of the cases was such a match found. Since the heuristic method could not be applied to the majority of the sequence pairs, it is also important to know how long such an unsuccessful search takes, after which it returns to the Needleman–Wunsch algorithm. On average, it took 0.023 s for each unsuccessful attempt to find a usable subalignment from the lookup table. This means that if we want to run the algorithm for all 4950 sequence pairs, the heuristic solution would take less time even if we add the extra times: the search phase, which took about 84 s; and the pre-processing time, which was around 319 s.

The same search phase with the fixed gap penalty was 0.07 s; multiplied by the average number of failed searches, this would mean 239 s of extra time. Adding the pre-processing phase, a total of about 393 s should be subtracted from the difference in run times, which is a little over a 6.5 min penalty, which is not really significant compared to the 2.5 h advantage.

Even with these extra times, the presented method was faster because the average difference between the two run times was around 2 and 9 h based on the chosen gap penalty.

## 5. Discussion

Based on the measured results, this heuristic method takes less time than the original dynamic programming algorithms and, with appropriate parameter setting, it provides a reasonable approximation of the optimal alignment. In the real data set with affine gap penalty, the heuristic approach found the optimal solution for every alignment in less time. The standard deviation of the alignment scores is strongly influenced by the length of the subalignments that have been stored and used. Measurements on a real data set have also shown that this method can be a favourable alternative to pairwise sequence alignment in a database environment. On the generated data sets, it took, on average, 50% less time, while on the Porcine circovirus samples, this ratio was around 75%, which in the case of affine gap penalty meant a 7 h difference in run time.

This heuristic approach can be combined with other heuristics, which can accelerate the global pairwise alignment; therefore, other methods containing this step can be performed in less time. This can increase the use of databases in the bioinformatics community. Among the heuristics described in Section 2, k-band alignment can be used in the implementation of our proposed method. Furthermore, the GLASS algorithm uses iterative *k* values to find an alignment close to the optimal one, and this approach can be applied to the technique presented in this paper.

Different methods can be developed to populate the lookup table. For example, the Smith–Waterman algorithm [36] can be used to determine the best local alignments on a sample set. Since we want to include in the table not just one subalignment but the most favourable ones, the original Smith–Waterman algorithm needs to be modified accordingly. The length of the resulting subalignments may vary, and it should also be taken into account that there are also subalignments that contain each other, so it may be necessary to set up additional filtering conditions among the local alignments above the threshold.

Two possible cases for scoring the alignments are implemented: fixed and affine gap penalty. However, similarity matrices can also be used. The presentation of the method focuses on DNA sequences, but the algorithm can also be applied to RNA sequences. In this variant, similarity matrices play a major role, for example, the ones based on mutation frequencies (PAM [37] and BLOSUM [38]).

It is a common approach to use BLAST to search a DNA sequence in a database. However, BLAST is a local alignment tool, and in this paper, we examined the problem of global alignments with a set of similar sequences. There are many use cases for global alignment: for example, it is possible to create phylogenetic trees based on the calculated distances, and it is the base of classical multiple sequence alignment.

In this study, the MonetDB column-based database management system provided the environment for the algorithm, but the method could be implemented in databases with other architectures. In order to optimize indexing, it may be useful to use a data structure that is favourable for solving pattern-matching problems (e.g., a suffix tree), since this is the most commonly performed search operation.

## 6. Conclusions

In this paper, we present a heuristic algorithm for the global sequence alignment task using the MonetDB columnar database management system and for when a large number of similar sequences need to be aligned. The key concept of the method is that relatively high-scoring subalignments generated from the sample set can be stored in the database and reused in subsequent alignments, thus creating a knowledge base. By identifying and inserting these subalignments, the problem size can be significantly reduced. Our approach is optimized for a database environment, which eliminates the need to query the database externally, thus reducing I/O overhead while providing all the benefits of a DBMS.

## Figures and Tables

**Table 1:** Parameters of the generated data sets.

Name	Average Length	Average Distance
h100	100	100
h200	200	100
h300	300	100
d50	100	50

**Table 2:** Average run time in seconds on different data sets depending on the length of the k-mers in the case of fixed gap penalty.

(**a**) d50 data set					(**b**) h100 data set				
k	***time*** ${}_{H}$	***time*** ${}_{O}$	***diff***	***diff*** ${}_{\%}$	k	***time*** ${}_{H}$	***time*** ${}_{O}$	***diff***	***diff*** ${}_{\%}$
5	0.026	0.05	0.024	48.0	5	0.026	0.05	0.024	48.0
7	0.027	0.05	0.023	46.0	7	0.029	0.051	0.022	43.14
10	0.028	0.05	0.022	44.0	10	0.029	0.051	0.022	43.14
15	0.028	0.051	0.023	45.1	15	0.028	0.052	0.024	46.15
(**c**) h200 data set					(**d**) h300 data set				
k	***time*** ${}_{H}$	***time*** ${}_{O}$	***diff***	***diff*** ${}_{\%}$	k	***time*** ${}_{H}$	***time*** ${}_{O}$	***diff***	***diff*** ${}_{\%}$
5	0.049	0.144	0.095	65.97	5	0.077	0.303	0.226	74.59
7	0.074	0.142	0.068	47.89	7	0.135	0.305	0.17	55.74
10	0.076	0.144	0.068	47.22	10	0.142	0.307	0.165	53.75
15	0.073	0.147	0.074	50.34	15	0.137	0.311	0.174	55.95

**Table 3:** Average run time in seconds on different data sets depending on the length of the k-mers in the case of affine gap penalty.

(**a**) d50 data set					(**b**) h100 data set				
k	***time*** ${}_{H}$	***time*** ${}_{O}$	***diff***	***diff*** ${}_{\%}$	k	***time*** ${}_{H}$	***time*** ${}_{O}$	***diff***	***diff*** ${}_{\%}$
5	0.043	0.108	0.065	60.19	5	0.05	0.114	0.064	56.14
7	0.04	0.108	0.068	62.96	7	0.048	0.114	0.066	57.89
10	0.045	0.108	0.063	58.33	10	0.05	0.113	0.063	55.75
15	0.046	0.108	0.062	57.41	15	0.05	0.113	0.063	55.75
(**c**) h200 data set					(**d**) h300 data set				
k	***time*** ${}_{H}$	***time*** ${}_{O}$	***diff***	***diff*** ${}_{\%}$	k	***time*** ${}_{H}$	***time*** ${}_{O}$	***diff***	***diff*** ${}_{\%}$
5	0.186	0.414	0.228	55.07	5	0.385	0.914	0.529	57.88
7	0.194	0.413	0.219	53.03	7	0.393	0.915	0.522	57.05
10	0.192	0.415	0.223	53.73	10	0.4	0.912	0.512	56.14
15	0.186	0.414	0.228	55.07	15	0.4	0.911	0.511	56.09

**Table 4:** Average pre-processing time in seconds depending on the size of the data set in the case of fixed gap penalty.

(**a**) d50 data set		(**b**) h100 data set	
size	time	size	time
10	1.6353	10	1.6355
15	3.9098	15	3.6893
20	7.2453	20	6.7533
(**c**) h200 data set		(**d**) h300 data set	
size	time	size	time
10	5.9945	10	13.627
15	13.725	15	32.5845
20	25.3698	20	59.5908

**Table 5:** Average pre-processing time in seconds depending on the size of the data set in the case of affine gap penalty.

(**a**) d50 data set		(**b**) h100 data set	
size	time	size	time
10	4.9605	10	4.6875
15	10.9318	15	10.7795
20	19.1918	20	19.603
(**c**) h200 data set		(**d**) h300 data set	
size	time	size	time
10	17.914	10	40.9673
15	41.4685	15	96.2643
20	75.712	20	177

**Table 6:** Time and peak memory usage of the proposed heuristic approach and a classical global pairwise algorithm for each data set.

(**a**) Average time per pairwise alignment in seconds.				
***method***	d50	h100	h200	h300
Bioconductor from file	0.0079	0.0079	0.0092	0.0092
Bioconductor from db	0.0457	0.0466	0.0472	0.0465
Proposed heuristic	0.028	0.029	0.076	0.142
(**b**) Maximum RAM usage during alignments in MB.				
***method***	d50	h100	h200	h300
Bioconductor from file	4.7	4.8	8.94	12.94
Bioconductor from db	5.86	6.26	9.82	13.82
Proposed heuristic	7.36	7.98	8.16	8.7

## Data Availability

Not applicable.

## Abbreviations

The following abbreviations are used in this manuscript:

BLOSUM	BLOcks Substitution Matrix
GLASS	GLobal Alignment SyStem
MUM	Maximal Unique Match
PAM	the unit of Accepted Point Mutations
